# Burden of Disease Due to Respiratory Syncytial Virus in Adults in Five Middle-Income Countries

**DOI:** 10.3390/idr16040057

**Published:** 2024-08-15

**Authors:** Jorge A. Gómez, Otavio Cintra, Arnas Berzanskis, Salma Pacheco, Henny Jaswantlal, Abdelkader El Hasnaoui, Desirée A. M. van Oorschot, Adriana Guzman-Holst

**Affiliations:** 1 Value Evidence & Outcomes, Vaccines, Emerging Markets, GSK, Buenos Aires 1429, Argentina; 2 Medical Affairs, Vaccines, Emerging Markets, GSK, Sao Paulo 04001-083, Brazil; otavio.a.cintra@gsk.com; 3 Medical Affairs, Vaccines, Emerging Markets, GSK, London WC1A 1DG, UK; arnas.x.berzanskis@gsk.com; 4 Medical Affairs, Vaccines, Emerging Markets, GSK, Mexico City 03330, Mexico; salma.r.pacheco@gsk.com; 5 Medical Affairs, Vaccines, Emerging Markets, GSK, Petaling Jaya 47800, Selangor, Malaysia; henny.x.jaswantlal@gsk.com; 6 Medical Affairs, Vaccines, Emerging Markets, GSK, Dubai 50199, United Arab Emirates; abdelkader.a.el-hasnaoui@gsk.com; 7 Medical Evidence Generation Emerging Markets, Vaccines GSK, 1300 Wavre, Belgium; desiree.x.van-oorschot@gsk.com (D.A.M.v.O.); adriana.x.guzman@gsk.com (A.G.-H.)

**Keywords:** respiratory syncytial virus, hospitalization, mortality, adult, case fatality, Latin America, Malaysia

## Abstract

Respiratory syncytial virus (RSV) is an important cause of severe respiratory disease in older adults. Understanding the disease burden is crucial for guiding vaccination policy and raising disease awareness. We estimated the burden of RSV hospitalizations and deaths in adults in five middle-income countries: Argentina, Brazil, Chile, Mexico, and Malaysia. Hospital discharge and death statistics due to any respiratory disease (ICD-10 codes: J00–99) from 2010 to 2022 were obtained. The RSV attributable burden on hospitalizations and deaths by age group was determined for 2019 using previously published estimates. Latin American countries showed distinct annual peaks in respiratory-related hospitalizations and deaths during winter months that were absent in Malaysia. Among ≥20-year-olds in 2019, there were 14,604 RSV-attributable hospitalizations nationally in Argentina, 44,323 in Brazil, 4529 in Chile, 7416 in Malaysia, and 8651 in Mexico, and 60–74% in ≥65-year-olds. There were also 3518 RSV-attributable deaths in Argentina, 9115 in Brazil, 801 in Chile, 704 in Malaysia, and 3806 in Mexico 79–88% in ≥65-year-olds. Incidences of RSV-attributable hospitalizations in ≥75-year-olds ranged between 256.3 and 294.3 per 100,000 population, and deaths between 33.6 and 112.9 per 100,000 population. RSV is associated with a substantial disease burden beyond pediatric age groups, and preventive vaccines could have a major impact on this burden, especially in older adults.

## 1. Introduction

Respiratory syncytial virus (RSV) is a common cause of severe respiratory disease in children and older adults. The risk of severe disease in adults is strongly age-dependent, with the highest hospitalization and mortality observed in adults over the age of 65 years [1,2]. In industrialized countries, the incidence of hospitalization for RSV in adults ≥65 years of age is 157 per 100,000 person-years, with an in-hospital fatality rate of 6.1% [3]. Risk factors for severe RSV disease are comorbid chronic or immunocompromising diseases [4,5,6]. Under-recognition is a significant issue due to symptom overlap with other viral infections [3], lack of awareness, and infrequent or delayed virology testing. Currently there is no specific therapy for RSV; therefore, there is a perception that a viral diagnosis will have no impact on the clinical management of cases. This leads to under-recognition and under-estimation of the disease burden.

The first RSV vaccines were approved in 2023 for the prevention of lower respiratory tract disease caused by RSV in adults 60 years of age and older [7,8]. Vaccine acceptance and high uptake will be contingent upon improving disease awareness amongst healthcare providers and the general public. In this regard, estimates of the disease burden will help to guide policy development by health authorities.

In high-income countries, a significant proportion of the severe RSV burden occurs in older adults, who account for 92% of RSV-associated hospitalizations and most deaths [9,10]. A modelling study conducted in the United Kingdom (UK) using national health data sources including laboratory results of viral testing, found that the burden of RSV was comparable to influenza in terms of general practitioner episodes (consultations), hospitalizations and deaths in adults aged ≥65 years [11]. Much less is known about the RSV disease burden in adults living in middle-income and low-income countries, although the available evidence points to a similar concentration of the burden in older adults [12,13,14]. The Global Burden of Disease Study reports higher attributable incidences of RSV than Influenza, as well as a higher burden of low respiratory tract infections in older adults than in pediatric populations in these countries [15,16].

We aimed to estimate the RSV burden in adults living in five middle-income countries using national hospital discharge and death statistics for any respiratory diseases (ICD-10 codes: J00–J99) available publicly or by request under information transparency law. In the absence of detailed RSV proportions by age and disease type in the analyzed countries, we used the attributable risk of RSV reported by Fleming et al. [11] for the UK to estimate the RSV burden.

A plain language statement is provided at the end of this article. An interactive graphical abstract is available as supplementary material (File S1).

## 2. Materials and Methods

### 2.1. Data Sources

Aggregate hospital discharge data and death certificate information for Argentina and Chile were requested from the respective Ministries of Health under data-sharing Transparency Laws. In Chile, the Ministry of Health database captures data for 100% of the population. In Argentina, the Hospital Discharge database covers only the hospitals in the public health sector (assisting approximately 36% of the population) [17], and the death certificate database captures all deaths in the country. Missing hospital discharge data observed during certain study years were supplemented as previously described and explained in the Supplement [18].

Similarly, the Brazilian Hospital Discharge database only captures information from hospitals in the public health system (assisting approximately 76.7% of the population) [19]. The death certificate database captures all deaths in the country. Data were obtained from the Unified Brazilian Health System (DATASUS) Hospital Morbidity Database and Mortality Information System [20].

In Mexico, the Mexican public health system covers approximately 80% of the population [21]. Hospital discharge and death certificate data were obtained from System of the Dynamic Analysis of Information (Cubos Dinamicos) from the Ministry of Health [22].

Hospital discharges and deaths data for Malaysia were obtained from the Malaysian Health Informatics Center (Pusat Informatik Kesihatan) operated by the planning division of the Ministry of Health, which captures data for 100% of the population [23].

Population data were obtained from official sources in each country [24,25,26,27,28].

The study used public and anonymous aggregate data. Ethics approval and patient consent were not applicable for this research and thus were not obtained.

### 2.2. Outcomes

The monthly number of hospital discharges and deaths with a primary diagnosis classified by International Classification of Diseases version 10 codes as any respiratory disease (ICD-10 codes: J00–J99) and age group were identified between January 2010 and December 2022. RSV-specific disease codes were not used for case identification because of the low rates of virological testing in middle-income countries.

Country data from 2019 were selected for the analysis of the RSV burden in order to have the latest available data prior to the coronavirus disease 2019 (COVID-19) pandemic. Data are presented for the following age group strata: 20–49 years, 50–64 years, 65–74 years, and ≥75 years, except for Chile, where data were available for adults aged 18–49 years instead of 20–49 years.

### 2.3. Statistical Analysis

Crude monthly incidences of hospitalization and monthly mortality due to any respiratory disease were calculated by age group from the national data sets using annual population estimates adjusted for the proportion of residents covered in the hospital discharges and deaths databases. The age-specific RSV attributable risk from any respiratory disease was derived from the modelling study of the RSV burden conducted in the UK and reported by Fleming et al., 2015 (Table 1) [11], who reported the RSV attributable risk by age group, by disease outcome (medical visit, hospitalization and death) and by certain ICD-10 code groups. The study by Fleming et al. 2015 was selected because of the robustness of the analysis and level of detail of the RSV data provided. Although the published study reported only a point estimate of different RSV attributable risks, we computed upper and lower levels of all attributable risks from other data reported in the same study. Fleming et al. 2015 reported RSV-attributable risks were applied to national hospitalization and death data considering similar age groups, health outcomes (hospitalization or death) and ICD-10 codes (codes for all respiratory diseases were considered, J00–J99) to estimate RSV burden in the five middle-income countries of interest.

Analyses were performed using Microsoft Excel for Microsoft 365 MSO (Version 2308; Microsoft Corporation, Redmond, WA, USA). 

## 3. Results

**Figure 1:**
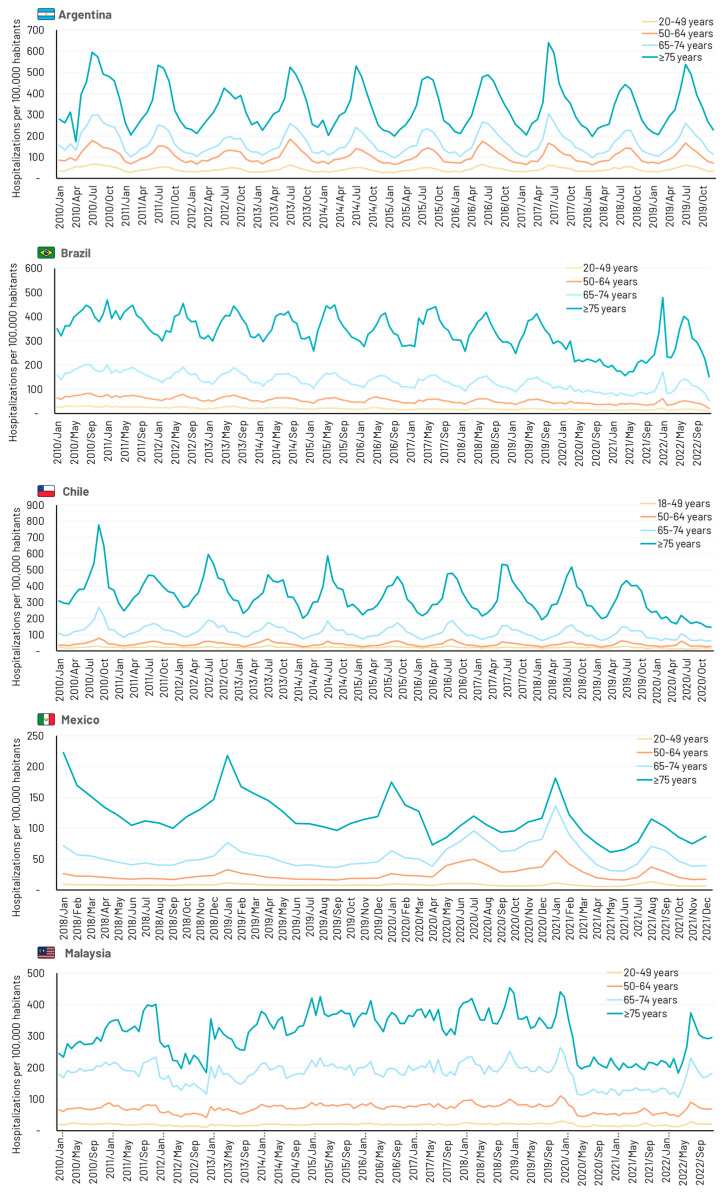
Time series showing monthly hospitalizations due to any respiratory disease (J00–J99) in five middle-income countries.

**Figure 2:**
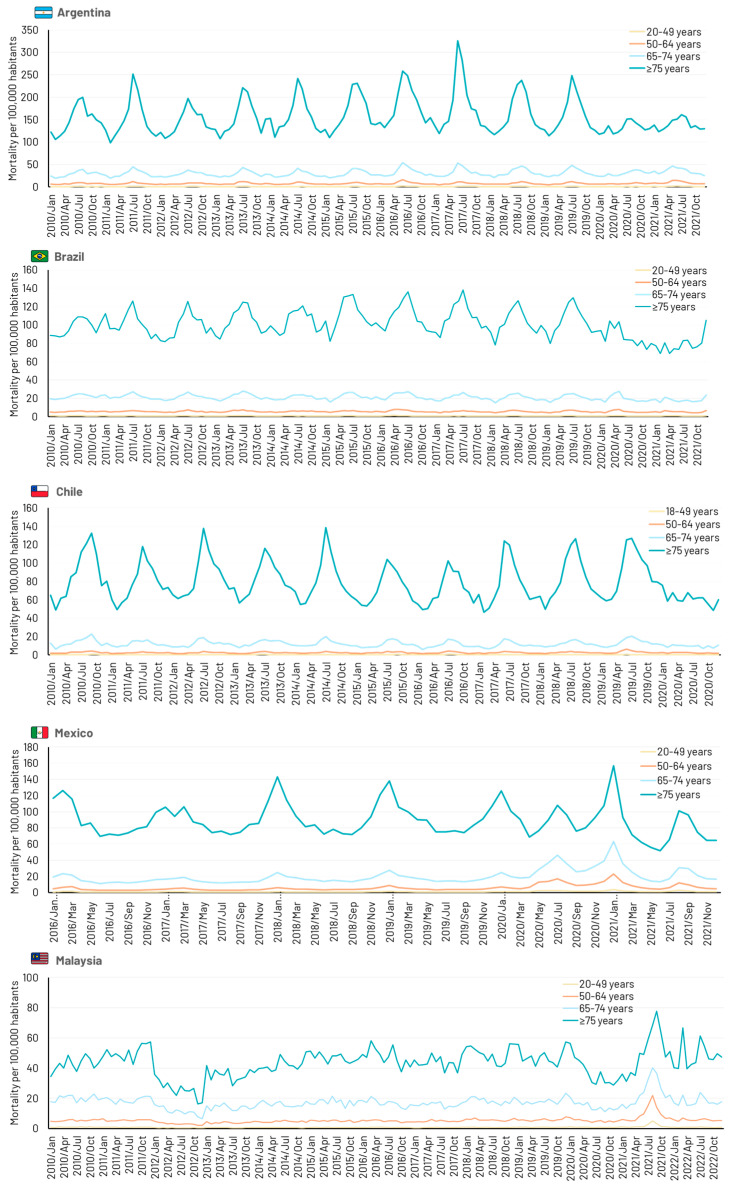
Time series showing monthly deaths due to any respiratory disease (J00–J99) in five middle-income countries.

All four Latin American countries showed distinct annual peaks in respiratory-related hospitalizations and deaths during northern or southern hemisphere winter months (Figure 1). Cyclical activity was absent in Malaysia where similar incidences of respiratory hospitalizations occurred all year round. The impact of the severe acute respiratory syndrome coronavirus 2 (SARS-CoV-2) pandemic is evident in countries with data extending through 2020, with marked decreases in hospitalizations reflecting the impact of lockdowns and social distancing. Deaths due to respiratory disease also decreased during the pandemic period, except in Malaysia, where a sharp increase in deaths was observed from mid-2021 in all age groups (Figure 2).

### 3.1. Argentina

**Figure 3:**
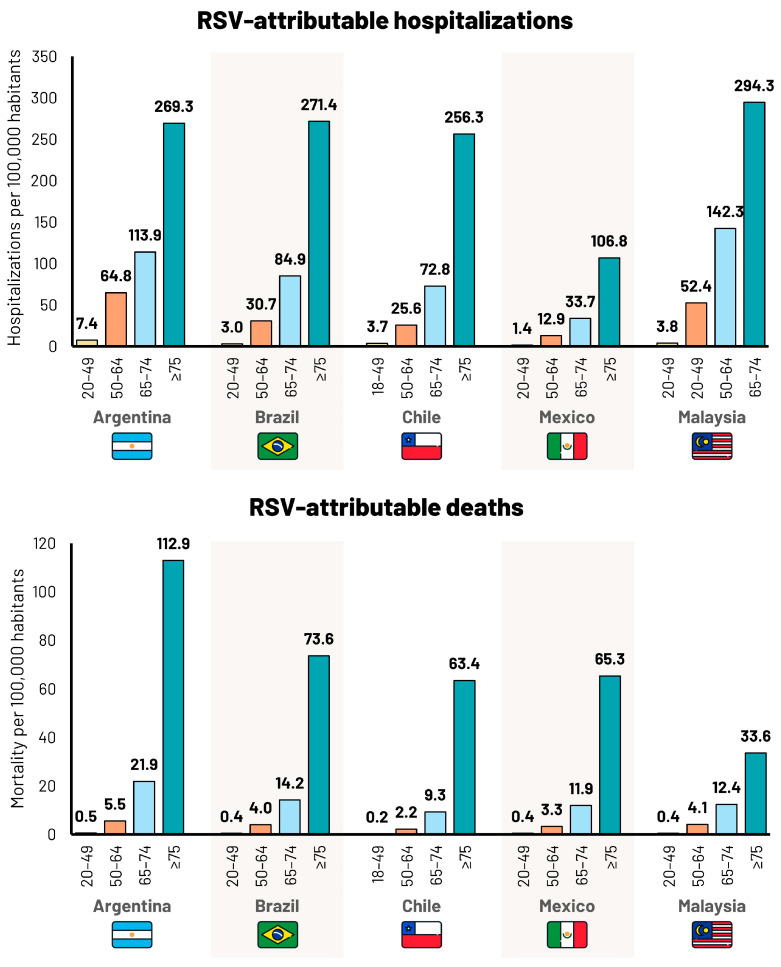
Incidence per 100,000 population of RSV-attributable hospitalizations and deaths per 100,000 population, by age group (years) and country (2019 data).

There were 1,073,139 hospital discharges with a primary diagnosis of any respiratory disease between 2010 and 2019 in the Argentinian Public Health sector (Table S1). The overall incidences per 100,000 population were 508 in 20–49-year-olds, 1257 in 50–64-year-olds, 2007 in 65–74-year-olds, and 4042 in ≥75-year-olds. Incidences tended to remain stable over the study period in all age groups. In 2019, the estimated number of hospitalizations attributed to RSV extrapolated to the total population was 14,604 (lower limit [LL] 11,205; upper limit [UL] 18,269), of which 63% were in ≥65-year-olds (Table 2). The incidence of hospitalizations attributed to RSV increased with age, from 7.4 per 100,000 population (LL 6.6; UL 11.1) in 20–49-year-olds, to 269.3 per 100,000 population (LL 207.4; UL 335.4) in ≥75-year-olds (Table 2, Figure 3).

There were 669,185 deaths due to any respiratory disease between 2010 and 2021 in Argentina, of which, 86% occurred in ≥65-year-olds, with an incidence of 84.6 per 100,000 (Table S2). In 2019, the estimated number of deaths attributed to RSV was 3518 (LL 2448; UL 4683), of which, 88% were ≥65-year-olds (Table 3). The incidence of deaths attributed to RSV increased with age from 0.5 per 100,000 population (LL 0.0; UL 1.1) in 20–49-year-olds, to 112.9 per 100,000 population (LL 78.8; UL 155.4) in ≥75-year-olds (Table 3, Figure 3).

### 3.2. Brazil

There were 8,708,898 hospital discharges with a primary diagnosis of any respiratory disease between 2010 and 2022 in the Brazilian public health sector (Table S1). The overall incidences per 100,000 population were 244 in 20–49-year-olds, 643 in 50–64-year-olds, 1563 in 65–74-year-olds, and 3940 in ≥75-year-olds. Incidences tended to remain stable over the study period. In 2019, the estimated number of hospitalizations attributed to RSV extrapolated to the total population was 44,323 (LL 33,857; UL 55,108), of which 71% were in ≥65-year-olds (Table 2). The incidence of hospitalizations attributed to RSV increased with age, from 3.0 per 100,000 population (LL 2.7; UL 4.5) in 20–49-year-olds to 271.4 per 100,000 population (LL 209.1; UL 338.0) in ≥75-year-olds (Table 2, Figure 3).

There were 1,675,021 deaths due to respiratory disease between 2010 and 2021 in Brazil. The overall incidences per 100,000 population were 10 in 20–49-year-olds, 68 in 50–64-year-olds, 252 in 65–74-year-olds, and 1201 in ≥75-year-olds (Table S2). In 2019, the estimated number of deaths attributed to RSV was 9115 (LL 6284; UL 12,129), of which, 82% were ≥65-year-olds (Table 3). The incidence of deaths attributed to RSV increased with age from 0.4 per 100,000 population (LL 0.0; UL 0.9) in 20–49-year-olds to 73.6 per 100,000 population (LL 51.4; UL 101.3) in ≥75-year-olds (Table 3, Figure 3).

### 3.3. Chile

There were 949,066 hospital discharges with a primary diagnosis of any respiratory disease between 2010 and 2020 in Chile (Table S1). The overall incidences per 100,000 population were 270 in 18–49-year-olds, 510 in 50–64-year-olds, 1367 in 65–74-year-olds, and 3980 in ≥75-year-olds. Incidences tended to decrease in all age groups over the study period. In 2019, the estimated number of hospitalizations attributed to RSV was 4529 (LL 3472; UL 5650), of which, 74% were ≥65-year-olds (Table 2). The incidence of hospitalizations attributed to RSV increased with age, from 3.7 per 100,000 population (LL 3.4; UL 5.6) in 18–49-year-olds to 256.3 per 100,000 population (LL 197.5; UL 319.2) in ≥75-year-olds (Table 2, Figure 3).

There were 115,865 deaths due to respiratory disease between 2010 and 2020 in Chile. The overall incidences per 100,000 population were in 18–49-year-olds, 31 in 50–64-year-olds, 144 in 65–74-year-olds, and 944 in ≥75-year-olds (Table S2). In 2019, the estimated number of deaths attributed to RSV was 801 (LL 559, UL 1070), of which, 89% were ≥65-year-olds (Table 3). The incidence of deaths attributed to RSV increased with age from 0.2 per 100,000 population (LL 0.0; UL 0.4) in 18–49-year-olds to 63.4 per 100,000 population (LL 44.2; UL 87.2) in ≥75-year-olds (Table 3, Figure 3).

### 3.4. Mexico

There were 630,082 hospital discharges with a primary diagnosis of any respiratory disease between 2018 and 2021 in Mexico (Table S1). The overall incidences per 100,000 population were 97 in 20–49-year-olds, 307 in 50–64-year-olds, 672 in 65–74-year-olds, and 141 in ≥75-year-olds, and tended to be stable over the study period. After analyzing the hospital discharge data obtained from official sources in Mexico, we concluded cases of any respiratory disease were most probably underreported. In 2019, the estimated number of hospitalizations attributed to RSV extrapolated to the total population was 8651 (LL 6638; UL 10,814), of which, 66% were ≥65-year-olds (Table 2). The incidence of hospitalizations attributed to RSV increased with age, from 1.4 per 100,000 population (LL 1.2; UL 2.1) in 20–49-year-olds to 106.8 per 100,000 population (LL 82.3; UL 133.1) in ≥75-year-olds (Table 2, Figure 3).

There were 422,873 deaths due to respiratory disease between 2016 and 2021 in Mexico. The overall incidences per 100,000 population were 13 in 20–49-year-olds, 73 in 50–64-year-olds, 243 in 65–74-year-olds, and 1069 in ≥75-year-olds (Table S2). There was a trend toward an increase in the incidence of respiratory deaths over time. In 2019, the estimated number of deaths attributed to RSV was 3806 (LL 2571; UL 5130), of which, 79% were ≥65-year-olds (Table 3). The incidence of deaths attributed to RSV increased with age from 0.4 per 100,000 population (LL 0.0; UL 0.9) in 20–49-year-olds to 65.3 per 100,000 population (LL 45.6; UL 89.9) in ≥75-year-olds (Table 3, Figure 3).

### 3.5. Malaysia

There were 1,230,600 hospital discharges with a primary diagnosis of any respiratory disease between 2010 and 2022 in Malaysia (Table S1). The overall incidences per 100,000 population were 235 in 20–49-year-olds, 901 in 50–64-year-olds, 2321 in 65–74-year-olds, and 4054 in ≥75-year-olds. There was considerable variation in incidence from year to year over the study period. In 2019, the estimated number of hospitalizations attributed to RSV was 7416 (LL 5645; UL 9208), of which, 60% were ≥65-year-olds (Table 2). The incidence of hospitalizations attributed to RSV increased with age, from 3.8 per 100,000 population (LL 3.4; UL 5.7) in 20–49-year-olds to 294.3 per 100,000 population (LL 226.7; UL 366.5) in ≥75-year-olds (Table 2, Figure 3).

There were 137,248 deaths due to respiratory disease between 2010 and 2022 in Malaysia. The overall incidences per 100,000 population were 10 in 20–49-year-olds, 66 in 50–64-year-olds, 209 in 65–74-year-olds, and 530 in ≥75-year-olds (Table S2). In 2019, the estimated number of deaths attributed to RSV was 704 (LL 468, UL 931), of which, 63% were ≥65-year-olds (Table 3). The incidence of deaths attributed to RSV increased with age from 0.4 per 100,000 population (LL 0.0; UL 1.0) in 20–49-year-olds to 33.6 per 100,000 population (LL 23.5; UL 46.3) in ≥75-year-olds (Table 3, Figure 3).

## 4. Discussion

This study applied published estimates for the RSV-attributable risk to national hospitalization and death data to obtain estimates of the RSV burden in five middle-income countries where the adult RSV burden is not well documented. Among ≥20-year-olds in 2019, we estimated 14,604 RSV hospitalizations nationally in Argentina, 44,323 in Brazil, 4529 in Chile, 8651 in Mexico, and 7416 in Malaysia. Individuals ≥65-year-olds accounted for 60–74% of all adult RSV-attributable hospitalizations in Latin American countries and Malaysia. In the same year, we estimated 3518, 9115, 801, 3806, and 704 deaths due to RSV in Argentina, Brazil, Chile, Mexico, and Malaysia, respectively, of which, 79–89% were ≥65-year-olds in Latin American countries versus 63% in Malaysia. Incidences of RSV hospitalizations and deaths were substantially higher in ≥65-year-olds versus other age groups. The incidence of RSV hospitalization in older adults was lower in our study than in a meta-analysis of United States (US) data, while RSV mortality was higher in our study than in a US analysis [29,30]. Conversely, estimates for both outcomes were well aligned with a meta-analysis conducted across high-income countries, and with the Fleming et al. study in the UK [10,11].

We observed the hospitalizations for any respiratory disease were substantially lower in Mexico than in other countries. This could reflect significant under-registration, and/or differences in healthcare-seeking behaviors, access to medical care, or clinical thresholds for hospitalization. The Ministry of Health hospital discharge database captures information from public and private hospitals and we consider that this lower level of hospitalization rates most probably originated as a problem of under-registration. This difference was not observed in the number of deaths identified in the Mexican database or in RSV mortality estimated for Mexico.

The data suggest a substantial burden of RSV in the countries studied, with a high disease burden in terms of hospitalizations and death in older adults aged 65+, which is consistent with patterns observed in high-income countries. Underlying risk factors such as chronic obstructive airway disease, asthma, and heart failure may increase this risk even more [31]. Results from the individual countries in our study are difficult to compare because of differences in healthcare systems, clinical practice and thresholds for hospitalization, and potential differences in ICD coding conventions and guidelines for coding from death certificates, or completeness of reporting. For example, the rate of hospitalizations in Argentina in ≥65-year-olds was not commensurate with the observed mortality rate, which could reflect ICD coding conventions on death certificates in Argentina.

Seasonal patterns of RSV outbreaks vary according to climate. Strong seasonal trends with peaks during winter months are typical in temperate climates but are not observed in tropical climates where RSV outbreaks may be related to rainy seasons [32,33]. Raw national data showed strong seasonality in the incidence of respiratory diseases in Argentina, Brazil, Chile and Mexico, which have mixed climates and include temperate and sub-tropical regions, and little impact of the season in tropical Malaysia. Similar seasonal patterns were observed in the RSV epidemiologic surveillance in these countries, including epidemiologic data in the pediatric population in Malaysia [34]. While national epidemiologic surveillance systems may be limited in geographic/population coverage and their capabilities to estimate the countrywide RSV burden, their reported virological seasonal data are aligned with the seasonality observed in our analysis [35,36,37].

Statistical modelling is a useful way to estimate the full burden of disease for infections where epidemiological surveillance data are lacking or incomplete [1]. Studies using methods similar to our own were used in other countries to this end, adding validity to our approach [38,39]. Our study provides important insights into the burden of RSV in adults living in five middle-income countries. A key strength is that the computed estimations for the RSV burden are not biased by the quality, or the sensitivity of viral diagnostic methods, the frequency of viral testing, or the type or quality of sample taken for viral testing. These limitations are typical of RSV surveillance and are one of the main constraints to identifying the full burden of RSV, with flow-on effects when raising awareness amongst clinicians and policy makers about the burden of RSV in older adults.

Potential limitations of our study are those associated with the use of healthcare databases that may be subject to incomplete reporting or incorrect coding practices that could impact disease estimates. Even though each country we studied captures 100% of deaths, translation of information on death certificates into ICD-10 codes may follow different conventions in different countries, further reducing their comparability. We only had access to primary diagnosis codes, and hospitalizations or deaths for other morbidities triggered or exacerbated by RSV were not captured, potentially leading to an underestimation of the disease burden. In addition, our study relies on the assumption that the RSV-attributable risk observed in the UK and reported by Fleming et al. is directly applicable to Latin American countries. Strong seasonality in respiratory diseases was similar in Latin American countries and the UK, suggesting that the application of UK data is likely appropriate in these settings, and the limited available data suggest similar epidemiology in Latin American countries versus the UK [11,40,41]. Nevertheless, the UK has a temperate climate and is geographically and ethnically distinct from Latin America and Asia, and the impact of these and other possible country-based factors on RSV epidemiology is not known. Furthermore, the study by Fleming et al. was subject to its own methodological limitations that may have produced inaccuracies in the attributable risks calculated. Our analysis shows Malaysia as a country without seasonality and a different age distribution of respiratory deaths compared to Latin America and our reference study from the UK [11]. Some COVID-19-related events could have contributed to the excess of mortality in Malaysia from mid-2021. However, we are uncertain how reporting guidelines developed by the different epidemiological surveillance systems may have impacted our data during this period.

RSV has a disproportionate effect on older adults, putting them at higher risk of severe outcomes (hospitalization and death). The burden of disease caused by influenza in older adults has long been recognized and global vaccination recommendations reflecting this burden have been in place for decades. RSV is deserving of similar attention, and prioritization of vaccination of older adults could have substantial impacts on healthcare resource utilization and budgets while contributing to healthy aging.

**Plain language statement**

*What is the context?*

- Respiratory syncytial virus (RSV) is an important cause of severe respiratory disease in older adults but may frequently go unrecognized.
- Understanding the burden of disease guides the use of preventative vaccines.

*What is new?*

- By using hospital and death statistics from Argentina, Brazil, Chile, Mexico, and Malaysia, this study showed large numbers of hospitalizations and deaths due to RSV in each country. Rates were highest in winter and were much higher in adults from the age of 65 years. Almost all RSV deaths occurred in this age group.

*What is the impact?*

- In view of the frequency and severity of RSV infections in older adults, prevention through vaccination should be prioritized.

## Figures and Tables

**Table 1:** RSV-attributable risk in adult populations reported by outcome, age group, and ICD-10 codes for the United Kingdom. Data from Fleming et al., 2015 [11].

	Hospital Discharges Due to Any Respiratory Diseases (J00–J99)			Deaths Due to Any Respiratory Diseases (J00–J99)		
Age Groups	% Attributable to RSV	Lower Limit	Upper Limit	% Attributable to RSV	Lower Limit	Upper Limit
18–49 years	1.40%	1.26%	2.10%	4.20%	0.00%	9.04%
50–64 years	5.20%	3.92%	6.41%	5.90%	4.72%	6.61%
65–74 years	5.80%	4.19%	6.83%	5.70%	4.18%	6.56%
≥75 years	6.80%	5.24%	8.47%	5.90%	4.12%	8.12%

ICD-10, International Classification of Diseases 10th revision; RSV, Respiratory syncytial virus. Note: the RSV-attributable risk by age in hospital discharges due to any respiratory disease and in deaths due to any respiratory disease were reported by Fleming et al., 2015. The lower and upper limits for those RSV-attributable risks were calculated based on data reported by Fleming et al., 2015.

**Table 2:** Incidence of hospitalizations due to any respiratory disease (J00–J99) and the RSV-attributable portion, by age group (years) and country (2019 data).

Country	Age Group (Years)	Population	Hospitalizations Due to Any Respiratory Disease (J00–J99)		RSV-Attributable Hospitalizations					
		Number ║	Number	Incidence/100,000	Number	LL	UL	Incidence/100,000 Population	LL	UL
Argentina *	20–49	6,878,499	36,253	527.0	1410	1269	2115	7.4	6.6	11.1
	50–64	2,234,244	27,845	1246.3	4022	3029	4957	64.8	48.8	79.9
	65–74	1,059,359	20,804	1963.8	3352	2423	3948	113.9	82.4	134.2
	≥75	778,069	30,811	3959.9	5820	4483	7248	269.3	207.4	335.4
	Total	10,950,171 *	115,713	1056.7	14,604	11,205	18,269	48.0	36.8	60.1
Brazil **	20–49	74,510,220	158,903	213.3	2900	2611	4352	3.0	2.7	4.5
	50–64	24,980,691	147,369	589.9	9991	7525	12,314	30.7	23.1	37.8
	65–74	9,400,404	137,632	1464.1	10,408	7525	12,259	84.9	61.4	100.0
	≥75	5,942,023	237,132	3990.8	21,023	16,196	26,183	271.4	209.1	338.0
	Total	114,833,339	681,036	593.1	44,323	33,857	55,108	29.6	22.6	36.8
Chile	18–49	9,085,578	24,300	267.5	340	306	510	3.7	3.4	5.6
	50–64	3,300,485	16,274	493.1	846	637	1043	25.6	19.3	31.6
	65–74	1,335,405	16,754	1254.6	972	703	1145	72.8	52.6	85.7
	≥75	924,817	34,860	3769.4	2370	1826	2952	256.3	197.5	319.2
	Total	14,646,285	92,188	629.4	4529	3472	5650	30.9	23.7	38.6
Mexico †	20–49	44,664,930	44,013	98.5	770	693	1156	1.4	1.2	2.1
	50–64	13,336,814	33,025	247.6	2147	1617	2646	12.9	9.7	15.9
	65–74	4,525,402	26,264	580.4	1904	1377	2243	33.7	24.3	39.6
	≥75	2,867,701	45,060	1571.3	3830	2951	4770	106.8	82.3	133.1
	Total	65,394,847	148,362	226.9	8651	6638	10,814	10.6	8.1	13.2
Malaysia	20–49	15,823,700	43,042	272.0	603	543	904	3.8	3.4	5.7
	50–64	4,558,200	45,925	1007.5	2388	1799	2943	52.4	39.5	64.6
	65–74	1,566,400	38,438	2453.9	2229	1612	2626	142.3	102.9	167.6
	≥75	746,100	32,288	4327.6	2196	1691	2734	294.3	226.7	366.5
	Total	22,694,400	159,693	703.7	7416	5645	9208	32.7	24.9	40.6

LL/UL, lower limit/upper limit of the computed number and incidence of RSV hospitalizations; RSV, Respiratory syncytial virus. * Hospitalizations due to any respiratory disease were divided by 0.36 to estimate the total number of RSV hospitalizations in the whole Argentine population; ** Hospitalizations due to any respiratory disease were divided by 0.767 to estimate the total number of RSV hospitalizations in the whole Brazilian population; † Hospitalizations due to any respiratory disease were divided by 0.80 to estimate the total number of RSV hospitalizations in the whole Mexican population ║ We divided the total Argentine, Brazilian and Mexican populations (by age group) by 0.36, 0.767 and 0.80, respectively, to compute the incidence/100,000, accounting for the target population covered in the hospital discharge database of each country.

**Table 3:** Mortality due to any respiratory disease (J00–J99) and RSV-attributable portion by age group (years) and country (2019 data).

Country	Age Group (Years)	Population	Deaths Due to Any Respiratory Disease (J00–J99)		RSV-Attributable Deaths					
			Number	Incidence/100,000	Number	LL	UL	Mortality/100,000	LL	UL
Argentina	20–49	19,106,941	2219	12	93	-	201	0.5	0.0	1.1
	50–64	6,206,232	5807	94	343	274	384	5.5	4.4	6.2
	65–74	2,942,665	11,288	384	643	471	741	21.9	16.0	25.2
	≥75	2,161,303	41,343	1913	2439	1703	3358	112.9	78.8	155.4
	Total	30,417,141	60,657	199	3518	2448	4683	11.6	8.0	15.4
Brazil	20–49	97,145,006	9243	10	388	-	836	0.4	0.0	0.9
	50–64	32,569,350	21,860	67	1290	1032	1445	4.0	3.2	4.4
	65–74	12,256,068	30,431	248	1735	1271	1997	14.2	10.4	16.3
	≥75	7,747,097	96,656	1248	5703	3981	7851	73.6	51.4	101.3
	Total	149,717,521	158,190	106	9115	6284	12,129	6.1	4.2	8.1
Chile	18–49	9,085,578	417	5	18	-	38	0.2	-	0.4
	50–64	3,300,485	1252	38	74	59	83	2.2	1.8	2.5
	65–74	1,335,405	2177	163	124	91	143	9.3	6.8	10.7
	≥75	924,817	9931	1074	586	409	807	63.4	44.2	87.2
	Total	14,646,285	13,777	94	801	559	1070	5.5	3.8	7.3
Mexico	20–49	55,831,163	5651	10	237	-	511	0.4	-	0.9
	50–64	16,671,018	9416	56	556	445	622	3.3	2.7	3.7
	65–74	5,656,752	11,813	209	673	493	775	11.9	8.7	13.7
	≥75	3,584,626	39,662	1106	2340	1633	3222	65.3	45.6	89.9
	Total	81,743,559	66,542	81	3806	2571	5130	4.7	3.1	6.3
Malaysia	20–49	15,823,700	1678	11	70	-	152	0.4	-	1.0
	50–64	4,558,200	3184	70	188	150	210	4.1	3.3	4.6
	65–74	1,566,400	3409	218	194	142	224	12.4	9.1	14.3
	≥75	746,100	4253	570	251	175	345	33.6	23.5	46.3
	Total	22,694,400	12,524	55	704	468	931	3.1	2.1	4.1

ICD-10, International Classification of Diseases 10th revision; LL/UL, lower limit/upper limit of the computed number and incidence of RSV deaths; N, number in the specified category; RSV, Respiratory syncytial virus. Respiratory disease deaths (ICD-10 codes: J00–J99) were reported based on death certificates for the entire population in each country (100% coverage). No assumptions were required to estimate RSV-attributable deaths in the whole population.

## Data Availability

Anonymized data and study documents can be requested for further research from https://www.gsk-studyregister.com/en/, accessed on 7 August 2024.

## Supplementary Materials

The following supporting information can be downloaded at: https://www.mdpi.com/article/10.3390/idr16040057/s1, Argentine Hospital Discharge data, Table S1: Number and incidence of hospitalizations for any respiratory disease (J00–J99) by age group (years) and country; Table S2: Number of deaths and mortality (per 100,000 population) due to any respiratory disease (J00–J99) by age group (years) and country; File S1: Interactive Graphical Abstract.
