# Supplementary material for: Burden of Disease Due to Respiratory Syncytial Virus in Adults in Five Middle-Income Countries
Source: Infect Dis Rep. 2024 Aug 15;16(4):750–62. doi: 10.3390/idr16040057 (PMC11354146; doi:10.3390/idr16040057)
Supplement: Supplementary file 1 [file idr-16-00057-s001.zip › File S1-Graphical abstract_Interactive version.pdf]

RESPIRATORY SYNCYTIAL VIRUS POSES A SUBSTANTIAL BURDEN TO OLDER ADULTS LIVING IN ARGENTINA, BRAZIL, CHILE, MEXICO, AND MALAYSIA

Respiratory Syncytial Virus (RSV) burden in adults is underestimated due to nonspecific symptomatology, limited surveillance, and lack of routine testing

Objective: estimation of **RSV burden** in adults in **5 middle-income countries** to inform **decision makers**

Monthly **hospitalizations** and **deaths** associated with any respiratory diseases (ICD-10 codes J00-99), were collected between 2010 – 2022

We applied the **age-specific RSV attributable risk on any respiratory disease** (ICD-10 codes J00-99), **age group** and **outcome** derived from an RSV study in the UK, to **estimate the burden of RSV in these countries for 2019**

Statistical modeling is a useful way to **estimate the full burden of disease** for infections in middle-income countries where epidemiological surveillance data are **lacking or incomplete**

This study suggests that **RSV imposes a significant burden on older adults** (≥65 years), and that **RSV preventive strategies like vaccines could help alleviate it**

ARGENTINA

Hospitalizations (per 100k) — Average: 48.0

Age (yo):	20-49	50-64	65-74	75+
	7.4	64.8	113.9	269.3

Deaths (per 100k) — Average: 11.6

Age (yo):	20-49	50-64	65-74	75+
	0.5	5.5	21.9	112.9

In Argentina, **1 out of 371 adults** aged 75 years and older are at risk of RSV hospitalization

CHILE

Hospitalizations (per 100k) — Average: 30.9

Age (yo):	18-49	50-64	65-74	75+
	3.7	25.6	72.8	256.3

Deaths (per 100k) — Average: 5.5

Age (yo):	18-49	50-64	65-74	75+
	0.2	2.2	9.3	63.4

In Chile, **1 out of 390 adults** aged 75 years and older are at risk of RSV hospitalization

MEXICO

Hospitalizations (per 100k) — Average: 10.6

Age (yo):	20-49	50-64	65-74	75+
	1.4	12.9	33.7	106.8

Deaths (per 100k) — Average: 4.7

Age (yo):	20-49	50-64	65-74	75+
	0.4	3.3	11.9	65.3

In Mexico, **1 out of 936 adults** aged 75 years and older are at risk of RSV hospitalization

BRAZIL

Hospitalizations (per 100k) — Average: 29.6

Age (yo):	20-49	50-64	65-74	75+
	3.0	30.7	84.9	271.4

Deaths (per 100k) — Average: 6.1

Age (yo):	20-49	50-64	65-74	75+
	0.4	4.0	14.2	73.6

In Brazil, **1 out of 368 adults** aged 75 years and older are at risk of RSV hospitalization

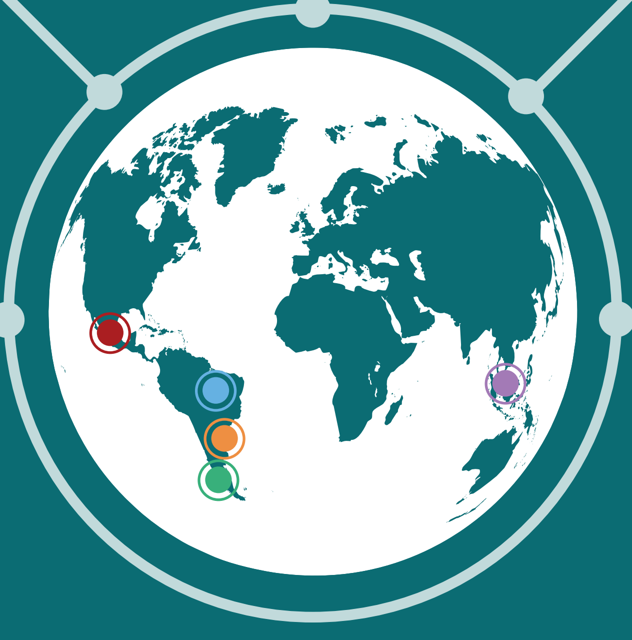

MALAYSIA

Hospitalizations (per 100k) — Average: 32.7

Age (yo):	20-49	50-64	65-74	75+
	3.8	52.4	142.3	294.3

Deaths (per 100k) — Average: 3.1

Age (yo):	20-49	50-64	65-74	75+
	0.4	4.1	12.4	33.6

In Malaysia, **1 out of 340 adults** aged 75 years and older are at risk of RSV hospitalization

ICD-10: International Classification of Diseases, 10th Revision; UK: United Kingdom; yo: years-old. ◄ Interactive link (click for more content).

RESPIRATORY SYNCYTIAL VIRUS POSES A SUBSTANTIAL BURDEN TO OLDER ADULTS LIVING IN ARGENTINA, BRAZIL, CHILE, MEXICO, AND MALAYSIA

Respiratory Syncytial Virus (RSV) burden in adults is underestimated due to nonspecific symptomatology, limited surveillance, and lack of routine testing

Objective: [illegible] **burden** in a[illegible] **middle-inc**[illegible] inform **deci**[illegible]

Monthly **ho**[illegible] **deaths** ass[illegible] respiratory [illegible] codes J00-[illegible] collected b[illegible] 2022

We applied the age-specific **RSV attributable risk on any respiratory disease** (ICD-10 codes J00-99), **age group** and **outcome** derived from an RSV study in the UK, to **estimate the burden of RSV in these countries for 2019**

Statistical modeling is a useful way to **estimate the full burden of disease** for infections in middle-income countries where epidemiological surveillance data are **lacking or incomplete**

This study suggests that **RSV imposes a significant burden on older adults** (≥65 years), and that **RSV preventive strategies like vaccines could help alleviate it**

ARGENTINA: annual hospitalizations and deaths

Hospitalizations — *All ages: 14,604*

Age (yo):	20-49	50-64	65-74	75+
Hospitalizations	1,410	4,022	3,352	5,820

Deaths — *All ages: 3,518*

Age (yo):	20-49	50-64	65-74	75+
Deaths	93	343	643	2,439

Clear seasonal pattern in the incidence of respiratory diseases (mixed climate).

CHILE

[illegible]pitalizations (per 100k)

Age (yo):	[illegible]	50-64	65-74	75+
Hospitalizations	[illegible]	25.6	72.8	256.3

[illegible] Deaths (per 100k)

Age (yo):	[illegible]	50-64	65-74	75+
Deaths	[illegible]	2.2	9.3	63.4

[illegible] **of 390 adults** aged 75 years and [illegible]sk of RSV hospitalization

MEXICO

Hospitalizations (per 100k) — *Average: 10.6*

Age (yo):	20-49	50-64	65-74	75+
Hospitalizations	1.4	12.9	33.7	106.8

Deaths (per 100k) — *Average: 4.7*

Age (yo):	20-49	50-64	65-74	75+
Deaths	0.4	3.3	11.9	65.3

In Mexico, **1 out of 936 adults** aged 75 years and older are at risk of RSV hospitalization

BRAZIL

Hospitalizations (per 100k) — *Average: 29.6*

Age (yo):	20-49	50-64	65-74	75+
Hospitalizations	3.0	30.7	84.9	271.4

Deaths (per 100k) — *Average: 6.1*

Age (yo):	20-49	50-64	65-74	75+
Deaths	0.4	4.0	14.2	73.6

In Brazil, **1 out of 368 adults** aged 75 years and older are at risk of RSV hospitalization

MALAYSIA

Hospitalizations (per 100k) — *Average: 32.7*

Age (yo):	20-49	50-64	65-74	75+
Hospitalizations	3.8	52.4	142.3	294.3

Deaths (per 100k) — *Average: 3.1*

Age (yo):	20-49	50-64	65-74	75+
Deaths	0.4	4.1	12.4	33.6

In Malaysia, **1 out of 340 adults** aged 75 years and older are at risk of RSV hospitalization

ICD-10: International Classification of Diseases, 10th Revision; UK: United Kingdom; yo: years-old. Interactive link (click for more content).

RESPIRATORY SYNCYTIAL VIRUS POSES A SUBSTANTIAL BURDEN TO OLDER ADULTS LIVING IN ARGENTINA, BRAZIL, CHILE, MEXICO, AND MALAYSIA

Respiratory Syncytial Virus (RSV) burden in adults is underestimated due to nonspecific symptomatology, limited surveillance, and lack of routine testing

Objective: estimation of **RSV burden** in adults in **5 middle-income countries** to inform **decision makers**

Monthly **hospitalizations** and **deaths** associated with any respiratory diseases (ICD-10 codes J00-99), were collected between 2010 - 2022

We applied the **age-specific RSV attributable risk on any respiratory disease** (ICD-10 codes J0[illegible] [illegible] age group and out[illegible] an RSV st[illegible] **estimate t[illegible] in these co[illegible]**

Statistical [illegible] useful way [illegible] **full burden** [illegible] infections i[illegible] countries w[illegible] epidemiolo[illegible] data are la[illegible] **incomplete**[illegible]

This study suggests that **RSV imposes a significant burden on older adults** (≥65 years), and that **RSV preventive strategies like vaccines could help alleviate it**

ARGENTINA

Hospitalizations (per 100k) — Average: 48.0

Age (yo):	20-49	50-64	65-74	75+
Hospitalizations	7.4	64.8	113.9	269.3

Deaths (per 100k) — Average: 11.6

Age (yo):	20-49	50-64	65-74	75+
Deaths	0.5	5.5	21.9	112.9

In Argentina, **1 out of 371 adults** aged 75 years and older are at risk of RSV hospitalization

CHILE

Hospitalizations (per 100k) — Average: 30.9

Age (yo):	18-49	50-64	65-74	75+
Hospitalizations	3.7	25.6	72.8	256.3

Deaths (per 100k) — Average: 5.5

Age (yo):	18-49	50-64	65-74	75+
Deaths	0.2	2.2	9.3	63.4

In Chile, **1 out of 390 adults** aged 75 years and older are at risk of RSV hospitalization

MEXICO

Hospitalizations (per 100k) — Average: 10.6

Age (yo):	20-49	50-64	65-74	75+
Hospitalizations	1.4	12.9	33.7	106.8

Deaths (per 100k) — Average: 4.7

Age (yo):	20-49	50-64	65-74	75+
Deaths	0.4	3.3	11.9	65.3

In Mexico, **1 out of 936 adults** aged 75 years and older are at risk of RSV hospitalization

BRAZIL

BRAZIL: annual hospitalizations and deaths

Hospitalizations — All ages: 44,322

Age (yo):	20-49	50-64	65-74	75+
Hospitalizations	2,900	9,991	10,408	21,023

Deaths — All ages: 9,116

Age (yo):	20-49	50-64	65-74	75+
Deaths	388	1,290	1,735	5,703

Clear seasonal pattern in the incidence of respiratory diseases (mixed climate).

MALAYSIA

Hospitalizations (per 100k) — Average: 32.7

Age (yo):	20-49	50-64	65-74	75+
Hospitalizations	3.8	52.4	142.3	294.3

Deaths (per 100k) — Average: 3.1

Age (yo):	20-49	50-64	65-74	75+
Deaths	0.4	4.1	12.4	33.6

In Malaysia, **1 out of 340 adults** aged 75 years and older are at risk of RSV hospitalization

ICD-10: International Classification of Diseases, 10th Revision; UK: United Kingdom; yo: years-old. Interactive link (click for more content).

RESPIRATORY SYNCYTIAL VIRUS POSES A SUBSTANTIAL BURDEN TO OLDER ADULTS LIVING IN ARGENTINA, BRAZIL, CHILE, MEXICO, AND MALAYSIA

Respiratory Syncytial Virus (RSV) burden in adults is underestimated due to nonspecific symptomatology, limited surveillance, and lack of routine testing

Objective: estimation of **RSV burden** in adults in **5 middle-income countries** to inform **decision makers**

Monthly **hospitalizations** and **deaths** associated with any respiratory diseases (ICD-10 codes J00-99), were collected between 2010 - 2022

2010
2022

We applied the **age-specific RSV attributable risk on any respiratory disease** (ICD-10 codes J00-99), **age group** and **outcome** derived from an RSV study in the UK, to **estimate the burden of RSV in these countries for 2019**

Statistical modeling is a useful way to **estimate the full burden of disease** for infections in middle-income countries where epidemiological surveillance data are **lacking or incomplete**

This study suggests that **RSV imposes a significant burden on older adults** (≥65 years), and that **RSV preventive strategies like vaccines could help alleviate it**

ARGENTINA

Hospita[illegible]

Average: 48.0

Age (yo):	20-49	50-64
	7.4	64.8

Death[illegible]

Average: 11.6

Age (yo):	20-49	50-64
	0.5	5.5

In Argentina, **1 out of 3[illegible]** and older are at risk of [illegible]

CHILE

CHILE: annual hospitalizations and deaths

Hospitalizations

All ages: 4,528

Age (yo):	18-49	50-64	65-74	75+
	340	846	972	2,370

Deaths

All ages: 802

Age (yo):	18-49	50-64	65-74	75+
	18	74	124	586

Clear seasonal pattern in the incidence of respiratory diseases (mixed climate).

MEXICO

[illegible]pitalizations (per 100k)

Age (yo):	50-64	65-74	75+
	12.9	33.7	106.8

Deaths (per 100k)

Age (yo):	50-64	65-74	75+
	3.3	11.9	65.3

[illegible]t of 936 adults aged 75 years [illegible] at risk of RSV hospitalization

BRAZIL

Hospitalizations (per 100k)

Average: 29.6

Age (yo):	20-49	50-64	65-74	75+
	3.0	30.7	84.9	271.4

Deaths (per 100k)

Average: 6.1

Age (yo):	20-49	50-64	65-74	75+
	0.4	4.0	14.2	73.6

In Brazil, **1 out of 368 adults** aged 75 years and older are at risk of RSV hospitalization

MALAYSIA

Hospitalizations (per 100k)

Average: 32.7

Age (yo):	20-49	50-64	65-74	75+
	3.8	52.4	142.3	294.3

Deaths (per 100k)

Average: 3.1

Age (yo):	20-49	50-64	65-74	75+
	0.4	4.1	12.4	33.6

In Malaysia, **1 out of 340 adults** aged 75 years and older are at risk of RSV hospitalization

ICD-10: International Classification of Diseases, 10th Revision; UK: United Kingdom; yo: years-old. *Interactive link (click for more content).*

RESPIRATORY SYNCYTIAL VIRUS POSES A SUBSTANTIAL BURDEN TO OLDER ADULTS LIVING IN ARGENTINA, BRAZIL, CHILE, MEXICO, AND MALAYSIA

Respiratory Syncytial Virus (RSV) burden in adults is underestimated due to nonspecific symptomatology, limited surveillance, and lack of routine testing

Objective: estimation of **RSV burden** in adults in **5 middle-income countries** to inform **decision makers**

Monthly **hospitalizations** and **deaths** associated with any respiratory diseases (ICD-10 codes J00-99), were collected between 2010 - 2022

We applied the **age-specific RSV attributable risk on any respiratory disease** (ICD-10 codes J00-99), **age group** and **outcome** derived from an RSV study in the UK, to **estimate the burden of RSV in these countries for 2019**

Statistical modeling is a useful way to **estimate the full burden of disease** for infections in middle-income countries where epidemiological surveillance data are **lacking or incomplete**

This study suggests that **RSV imposes a significant burden on older adults** (≥65 years), and that **RSV preventive strategies like vaccines could help alleviate it**

ARGENTINA

Hospitalizations (per 100k) — *Average: 48.0*

Age (yo):	20-49	50-64	65-74	75+
	7.4	64.8	113.9	269.3

Deaths (per 100k) — *Average: 11.6*

Age (yo):	20-49	50-64	65-74	75+
	0.5	5.5	21.9	112.9

In Argentina, **1 out of 371 adults** aged 75 years and older are at risk of RSV hospitalization

CHILE

MEXICO

MEXICO: annual hospitalizations and deaths

Hospitalizations — *All ages: 8,651*

Age (yo):	20-49	50-64	65-74	75+
	770	2,147	1,904	3,830

Deaths — *All ages: 3,806*

Age (yo):	20-49	50-64	65-74	75+
	237	556	673	2,340

Clear seasonal pattern in the incidence of respiratory diseases (mixed climate).

BRAZIL

Hospitalizations (per 100k) — *Average: 29.6*

Age (yo):	20-49	50-64	65-74	75+
	3.0	30.7	84.9	271.4

Deaths (per 100k) — *Average: 6.1*

Age (yo):	20-49	50-64	65-74	75+
	0.4	4.0	14.2	73.6

In Brazil, **1 out of 368 adults** aged 75 years and older are at risk of RSV hospitalization

MALAYSIA

Hospitalizations (per 100k) — *Average: 32.7*

Age (yo):	20-49	50-64	65-74	75+
	3.8	52.4	142.3	294.3

Deaths (per 100k) — *Average: 3.1*

Age (yo):	20-49	50-64	65-74	75+
	0.4	4.1	12.4	33.6

In Malaysia, **1 out of 340 adults** aged 75 years and older are at risk of RSV hospitalization

ICD-10: International Classification of Diseases, 10th Revision; UK: United Kingdom; yo: years-old. Interactive link (click for more content).

RESPIRATORY SYNCYTIAL VIRUS POSES A SUBSTANTIAL BURDEN TO OLDER ADULTS LIVING IN ARGENTINA, BRAZIL, CHILE, MEXICO, AND MALAYSIA

Respiratory Syncytial Virus (RSV) burden in adults is underestimated due to nonspecific symptomatology, limited surveillance, and lack of routine testing

Objective: estimation of **RSV burden** in adults in **5 middle-income countries** to inform **decision makers**

Monthly **hospitalizations** and **deaths** associated with any respiratory diseases (ICD-10 codes J00-99), were collected between 2010 - 2022

We applied the **age-specific RSV attributable risk on any respiratory disease** (ICD-10 codes J00-99), **age group** and **outcome** derived from an RSV study in the UK, to **estimate the burden of RSV in these countries for 2019**

Statistical modeling is a useful way to **estimate the full burden of disease** for infections in middle-income countries where epidemiological surveillance data are **lacking or incomplete**

This study suggests that **RSV imposes a significant burden on older adults** (≥65 years), and that **RSV preventive strategies like vaccines could help alleviate it**

ARGENTINA

Hospitalizations (per 100k) — Average: 48.0

Age (yo):	20-49	50-64	65-74	75+
Hospitalizations	7.4	64.8	113.9	269.3

Deaths (per 100k) — Average: 11.6

Age (yo):	20-49	50-64	65-74	75+
Deaths	0.5	5.5	21.9	112.9

In Argentina, **1 out of 371 adults** aged 75 years and older are at risk of RSV hospitalization

CHILE

Hospitalizations (per 100k) — Average: 30.9

Age (yo):	18-49	50-64	65-74	75+
Hospitalizations	3.7	25.6	72.8	256.3

Deaths (per 100k) — Average: 5.5

Age (yo):	18-49	50-64	65-74	75+
Deaths	0.2	2.2	9.3	63.4

In Chile, **1 out of 390 adults** aged 75 years and older are at risk of RSV hospitalization

MEXICO

Hospitalizations (per 100k) — Average: 10.6

Age (yo):	20-49	50-64	65-74	75+
Hospitalizations	1.4	12.9	33.7	106.8

Deaths (per 100k) — Average: 4.7

Age (yo):	20-49	50-64	65-74	75+
Deaths	0.4	3.3	11.9	65.3

In Mexico, **1 out of 936 adults** aged 75 years and older are at risk of RSV hospitalization

BRAZIL

Hospitalizations (per 100k) — Average: 29.6

Age (yo):	20-49	50-64	65-74	75+
Hospitalizations	3.0	30.7	84.9	271.4

Deaths (per 100k) — Average: 6.1

Age (yo):	20-49	50-64	65-74	75+
Deaths	0.4	4.0	14.2	73.6

In Brazil, **1 out of 368 adults** aged 75 years and older are at risk of RSV hospitalization

MALAYSIA

MALAYSIA: annual hospitalizations and deaths

Hospitalizations — All ages: 7,416

Age (yo):	20-49	50-64	65-74	75+
Hospitalizations	603	2,388	2,229	2,196

Deaths — All ages: 703

Age (yo):	20-49	50-64	65-74	75+
Deaths	70	188	194	251

Little impact of season in the incidence of respiratory diseases (tropical climate).

ICD-10: International Classification of Diseases, 10th Revision; UK: United Kingdom; yo: years-old. Interactive link (click for more content).
