# Supplementary material for: Burden of Disease Due to Respiratory Syncytial Virus in Adults in Five Middle-Income Countries
Source: Infect Dis Rep. 2024 Aug 15;16(4):750–62. doi: 10.3390/idr16040057 (PMC11354146; doi:10.3390/idr16040057)
Supplement: Supplementary file 1 [file idr-16-00057-s001.zip › Supplementary tables.pdf]

Burden of Disease Due to Respiratory Syncytial Virus in Adults in Five Middle-Income Countries

Jorge A. Gómez *, Otavio Cintra, Arnas Berzanskis, Salma Pacheco, Henny Jaswantlal, Abdelkader El Hasnaoui, Desirée A. M. van Oorschot and Adriana Guzman-Holst

*Correspondence: jorge.a.gomez@gsk.com; Tel.: +54-911-4070-7401

SUPPLEMENT

Argentine Hospital Discharge data

The letter received by the Ministry of Health in response to our request (based on the transparency law) mentions that a few provinces had missing data in the hospital discharge database for certain years in the period requested. We have completed those missing data with the latest data on hospital discharges reported by the same province. In all, we have received 1,000,435 reports of hospital discharges associated with any respiratory diseases (ICD-codes: J00–J99) between 2010–2019 from the Argentine Public Health sector. After missing data was completed as mentioned, the total number of hospital discharges identified increased by 7% over the entire period and by 6% in 2019.

Data Request Register – Malaysia

In Malaysia, the data request was registered under the National Medical Research Register (NMRR), ID number ID-24-00529-QDY.

Table S1 Number and incidence of hospitalizations for any respiratory disease (J00–J99) by age group (years) and country

ARGENTINA (2010–2019)

Age group	2010	2011	2012	2013	2014	2015	2016	2017	2018	2019	Total
**Reported number of hospitalizations due to any respiratory disease (ICD-10 codes: J00–J99)**											
20–49	38,678	30,242	29,119	31,201	30,021	30,158	35,566	34,917	33,777	36,253	329,932
50–64	29,994	25,554	24,673	27,393	25,086	24,907	28,591	27,209	25,739	27,845	266,991
65–74	21,797	16,941	15,962	17,947	17,810	17,991	20,752	21,216	18,929	20,804	190,149
≥75	31,636	26,541	25,452	27,819	27,261	27,268	29,766	31,765	27,748	30,811	286,067
Total	122,105	99,278	95,206	104,360	100,178	100,324	114,675	115,107	106,193	115,713	1,073,139
**Total population covered by the hospital discharge database (36%)**											
20–49	6,082,856	6,172,922	6,264,622	6,357,114	6,449,464	6,540,822	6,630,298	6,716,981	6,799,997	6,878,499	64,893,575
50–64	2,004,502	2,034,672	2,063,296	2,090,402	2,116,043	2,140,336	2,163,447	2,185,986	2,209,136	2,234,244	21,242,064
65–74	840,095	860,485	883,252	907,728	933,146	958,909	984,691	1,010,278	1,035,281	1,059,359	9,473,223
≥75	651,431	661,793	672,461	683,769	696,053	709,567	724,384	740,575	758,392	778,069	7,076,493
Total	9,578,884	9,729,871	9,883,630	10,039,013	10,194,706	10,349,634	10,502,821	10,653,819	10,802,805	10,950,171	102,685,355
**Incidence of hospitalizations due to any respiratory disease per 100,000 population**											
20–49	636	490	465	491	465	461	536	520	497	527	508
50–64	1,496	1,256	1,196	1,310	1,186	1,164	1,322	1,245	1,165	1,246	1,257
65–74	2,595	1,969	1,807	1,977	1,909	1,876	2,107	2,100	1,828	1,964	2,007
≥75	4,856	4,010	3,785	4,068	3,917	3,843	4,109	4,289	3,659	3,960	4,042
Total	1,275	1,020	963	1,040	983	969	1,092	1,080	983	1,057	1,045

BRAZIL (2010–2022)

Age group	2010	2011	2012	2013	2014	2015	2016	2017	2018	2019	2020	2021	2022	Total
**Reported number of hospitalizations due to any respiratory disease (ICD-10 codes: J00–J99)**														
20–49	238,963	225,379	208,001	205,945	192,651	174,135	169,511	158,167	159,625	158,903	139,229	132,411	140,122	2,303,042
50–64	168,149	166,785	156,893	159,910	151,918	148,739	152,746	148,206	147,733	147,369	126,461	121,942	134,515	1,931,366
65–74	142,190	142,970	131,997	136,181	131,589	133,224	131,076	138,827	135,088	137,632	109,799	104,641	138,216	1,713,430
≥75	201,283	213,683	200,444	211,411	214,378	224,533	214,027	238,892	231,367	237,132	171,687	160,238	241,985	2,761,060
Total	750,585	748,817	697,335	713,447	690,536	680,631	667,360	684,092	673,813	681,036	547,176	519,232	654,838	8,708,898
**Total population covered by the hospital discharge database (76.7%)**														
20–49	69,044,232	69,778,632	70,447,150	71,067,643	71,667,933	72,264,422	72,852,751	73,437,826	74,001,939	74,510,220	74,940,940	75,306,528	75,560,155	944,880,370
50–64	19,375,247	20,028,858	20,681,942	21,331,076	21,972,689	22,603,641	23,222,568	23,830,022	24,419,357	24,980,691	25,509,507	26,006,773	26,470,425	300,432,796
65–74	6,671,389	6,891,394	7,128,243	7,385,314	7,666,358	7,972,739	8,302,158	8,653,351	9,021,653	9,400,404	9,785,315	10,175,508	10,568,784	109,622,610
≥75	4,274,609	4,430,489	4,603,003	4,785,479	4,969,888	5,152,259	5,337,062	5,526,815	5,726,094	5,942,023	6,178,524	6,436,224	6,716,795	70,079,264
Total	99,365,477	101,129,373	102,860,338	104,569,512	106,276,868	107,993,061	109,714,539	111,448,014	113,169,043	114,833,339	116,414,285	117,925,033	119,316,159	1,425,015,039
**Incidence of hospitalizations due to any respiratory disease per 100,000 population**														
20–49	346	323	295	290	269	241	233	215	216	213	186	176	185	244
50–64	868	833	759	750	691	658	658	622	605	590	496	469	508	643
65–74	2,131	2,075	1,852	1,844	1,716	1,671	1,579	1,604	1,497	1,464	1,122	1,028	1,308	1,563
≥75	4,709	4,823	4,355	4,418	4,314	4,358	4,010	4,322	4,041	3,991	2,779	2,490	3,603	3,940
Total	755	740	678	682	650	630	608	614	595	593	470	440	549	611

CHILE (2010–2020)

Age group	2010	2011	2012	2013	2014	2015	2016	2017	2018	2019	2020	Total
**Reported number of hospitalizations due to any respiratory disease (ICD-10 codes: J00–J99)**												
18–49	23,744	23,337	23,632	25,597	22,950	24,467	24,123	22,109	23,507	24,300	19,096	256,862
50–64	15,452	14,661	15,025	16,309	14,031	15,322	16,325	15,091	15,626	16,274	14,028	168,144
65–74	16,460	14,752	15,678	15,724	14,663	15,394	15,913	16,017	16,155	16,754	12,323	169,833
≥75	35,221	30,960	34,129	31,764	31,535	31,324	32,656	35,400	34,889	34,860	21,489	354,227
Total	90,877	83,710	88,464	89,394	83,179	86,507	89,017	88,617	90,177	92,188	66,936	949,066
**Total population covered by the hospital discharge database (100%)**												
18–49	8,251,393	8,332,294	8,407,167	8,463,306	8,516,650	8,566,336	8,624,370	8,733,996	8,899,268	9,085,578	9,264,432	95,144,790
50–64	2,588,219	2,675,332	2,762,524	2,848,603	2,935,663	3,017,783	3,090,645	3,161,019	3,234,831	3,300,485	3,359,295	32,974,399
65–74	920,338	952,502	987,847	1,024,819	1,061,928	1,104,041	1,155,332	1,211,108	1,273,583	1,335,405	1,397,997	12,424,900
≥75	678,646	701,320	724,869	749,534	775,386	802,322	830,386	859,688	891,612	924,817	960,619	8,899,199
Total	12,438,596	12,661,448	12,882,407	13,086,262	13,289,627	13,490,482	13,700,733	13,965,811	14,299,294	14,646,285	14,982,343	149,443,288
**Incidence of hospitalizations due to any respiratory disease per 100,000 population**												
18–49	288	280	281	302	269	286	280	253	264	267	206	270
50–64	597	548	544	573	478	508	528	477	483	493	418	510
65–74	1,788	1,549	1,587	1,534	1,381	1,394	1,377	1,323	1,268	1,255	881	1,367
≥75	5,190	4,415	4,708	4,238	4,067	3,904	3,933	4,118	3,913	3,769	2,237	3,980
Total	731	661	687	683	626	641	650	635	631	629	447	635

MEXICO (2018–2021)

Age group	2018	2019	2020	2021	Total
**Reported number of hospitalizations due to any respiratory disease (ICD-10 codes: J00–J99)**					
20–49	42,961	44,013	45,058	42,826	174,858
50–64	31,766	33,025	54,792	46,536	166,119
65–74	25,935	26,264	38,160	33,984	124,343
≥75	45,138	45,060	39,746	34,818	164,762
Total	145,800	148,362	177,756	158,164	630,082
**Total population covered by the hospital discharge database (80%)**					
20–49	44,227,408	44,664,930	45,071,081	45,450,374	179,413,793
50–64	12,935,206	13,336,814	13,735,881	14,131,783	54,139,685
65–74	4,353,861	4,525,402	4,708,518	4,903,038	18,490,818
≥75	2,782,785	2,867,701	2,957,267	3,051,785	11,659,538
Total	64,299,260	65,394,847	66,472,746	67,536,980	263,703,834
**Incidence of hospitalizations due to respiratory disease per 100,000 population**					
20–49	97	99	100	94	97
50–64	246	248	399	329	307
65–74	596	580	810	693	672
≥75	1,622	1,571	1,344	1,141	1,413
Total	227	227	267	234	239

MALAYSIA (2010–2022)

Age group	2010	2011	2012	2013	2014	2015	2016	2017	2018	2019	2020	2021	2022	Total
**Reported number of hospitalizations due to any respiratory disease (ICD-10 codes: J00–J99)**														
20–49	33,276	32,808	23,889	28,478	33,652	36,503	36,720	36,259	41,329	43,042	33,514	31,891	37,309	345,956
50–64	27,362	30,878	22,311	28,908	34,833	38,866	38,280	40,307	45,643	45,925	33,552	32,567	38,522	353,313
65–74	21,868	24,633	18,340	23,534	27,188	30,783	30,402	33,099	37,694	38,438	28,129	26,638	35,278	285,979
≥75	15,799	20,966	14,560	20,180	24,086	27,927	27,971	28,858	32,717	32,288	23,433	20,950	28,900	245,352
Total	98,305	109,285	79,100	101,100	119,759	134,079	133,373	138,523	157,383	159,693	118,628	112,046	140,009	1,230,600
**Total population covered by the hospital discharge database (100%)**														
20–49	13,243,900	13,657,800	14,043,300	14,395,400	14,711,400	14,987,000	15,218,700	15,436,700	15,637,200	15,823,700	15,998,500	16,186,500	16,356,600	147,155,100
50–64	3,260,300	3,415,600	3,565,100	3,711,000	3,855,500	3,999,000	4,139,000	4,280,800	4,421,400	4,558,200	4,690,700	4,820,100	4,947,600	39,205,900
65–74	947,300	999,500	1,056,000	1,115,700	1,180,400	1,249,900	1,323,100	1,401,400	1,482,800	1,566,400	1,652,400	1,739,500	1,827,600	12,322,500
≥75	477,800	496,300	524,900	557,100	589,700	620,700	650,400	679,500	710,200	746,100	790,300	841,700	900,700	6,052,700
Total	17,929,300	18,569,200	19,189,300	19,779,200	20,337,000	20,856,600	21,331,200	21,798,400	22,251,600	22,694,400	23,131,900	23,587,800	24,032,500	204,736,200
**Incidence of hospitalizations due to respiratory disease per 100,000 population**														
20–49	251	240	170	198	229	244	241	235	264	272	209	197	228	235
50–64	839	904	626	779	903	972	925	942	1,032	1,008	715	676	779	901
65–74	2,308	2,465	1,737	2,109	2,303	2,463	2,298	2,362	2,542	2,454	1,702	1,531	1,930	2,321
≥75	3,307	4,224	2,774	3,622	4,084	4,499	4,301	4,247	4,607	4,328	2,965	2,489	3,209	4,054
Total	548	589	412	511	589	643	625	635	707	704	513	475	583	601

ICD-10, International Classification of Diseases 10th revision

Table S2 Number of deaths and mortality (per 100,000 population) due to any respiratory disease (J00–J99) by age group (years) and country

ARGENTINA (2010–2021)

Age group	2010	2011	2012	2013	2014	2015	2016	2017	2018	2019	2020	2021	Total
**Reported number of deaths due to any respiratory disease (ICD-10 codes: J00-J99)**													
20–49	2,131	2,028	2,054	2,132	1,940	1,979	2,707	2,273	2,367	2,219	2,186	2,972	26,988
50–64	5,068	5,067	5,085	5,577	5,290	5,336	6,440	5,755	5,733	5,807	5,612	7,458	68,228
65–74	8,085	8,367	8,469	8,862	8,860	9,566	11,177	10,946	11,282	11,288	10,900	13,026	120,828
≥75	32,472	33,077	32,507	35,064	36,921	38,888	43,692	44,564	41,148	41,343	35,185	38,280	453,141
Total	47,756	48,539	48,115	51,635	53,011	55,769	64,016	63,538	60,530	60,657	53,883	61,736	669,185
**Total Population**													
20–49	16,896,822	17,147,005	17,401,727	17,658,650	17,915,179	18,168,951	18,417,494	18,658,281	18,888,880	19,106,941	19,310,294	19,497,839	219,068,063
50–64	5,568,062	5,651,866	5,731,377	5,806,673	5,877,897	5,945,379	6,009,576	6,072,183	6,136,488	6,206,232	6,284,557	6,373,043	71,663,333
65–74	2,333,596	2,390,235	2,453,478	2,521,468	2,592,072	2,663,635	2,735,253	2,806,327	2,875,780	2,942,665	3,006,157	3,065,803	32,386,469
≥75	1,809,531	1,838,313	1,867,946	1,899,357	1,933,481	1,971,019	2,012,179	2,057,152	2,106,645	2,161,303	2,221,565	2,287,469	24,165,960
Total	26,608,011	27,027,419	27,454,528	27,886,148	28,318,629	28,748,984	29,174,502	29,593,943	30,007,793	30,417,141	30,822,573	31,224,154	347,283,825
**Incidence of deaths due to any respiratory disease per 100,000 population**													
20–49	13	12	12	12	11	11	15	12	13	12	11	15	12
50–64	91	90	89	96	90	90	107	95	93	94	89	117	95
65–74	346	350	345	351	342	359	409	390	392	384	363	425	373
≥75	1,794	1,799	1,740	1,846	1,910	1,973	2,171	2,166	1,953	1,913	1,584	1,673	1,875
Total	179	180	175	185	187	194	219	215	202	199	175	198	193

BRAZIL (2010–2021)

Age group	2010	2011	2012	2013	2014	2015	2016	2017	2018	2019	2020	2021	Total
**Reported number of deaths due to any respiratory disease (ICD-10 codes: J00–J99)**													
20–49	9,608	9,352	9,597	10,340	9,375	9,480	10,641	8,780	9,246	9,243	9,965	9,915	115,542
50–64	16,954	18,130	18,051	19,551	19,397	20,154	22,989	20,428	21,090	21,860	22,096	21,640	242,340
65–74	22,938	23,788	23,792	25,598	25,246	27,257	29,144	28,694	29,187	30,431	30,184	29,132	325,391
≥75	65,080	70,732	71,250	77,649	80,795	88,827	91,140	94,105	91,905	96,656	84,259	79,350	991,748
Total	114,580	122,002	122,690	133,138	134,813	145,718	153,914	152,007	151,428	158,190	146,504	140,037	1,675,021
**Total population**													
20–49	90,018,555	90,976,052	91,847,653	92,656,640	93,439,287	94,216,978	94,984,030	95,746,840	96,482,319	97,145,006	97,706,571	98,183,218	1,133,403,149
50–64	25,261,078	26,113,244	26,964,722	27,811,051	28,647,574	29,470,197	30,277,142	31,069,129	31,837,493	32,569,350	33,258,809	33,907,135	357,186,924
65–74	8,698,030	8,984,868	9,293,668	9,628,832	9,995,251	10,394,705	10,824,196	11,282,074	11,762,260	12,256,068	12,757,907	13,266,634	129,144,493
≥75	5,573,154	5,776,387	6,001,308	6,239,216	6,479,645	6,717,417	6,958,360	7,205,756	7,465,572	7,747,097	8,055,442	8,391,426	82,610,780
Total	129,550,817	131,850,551	134,107,351	136,335,739	138,561,757	140,799,297	143,043,728	145,303,799	147,547,644	149,717,521	151,778,729	153,748,413	1,702,345,346
**Incidence of deaths due to any respiratory disease per 100,000 population**													
20–49	11	10	10	11	10	10	11	9	10	10	10	10	10
50–64	67	69	67	70	68	68	76	66	66	67	66	64	68
65–74	264	265	256	266	253	262	269	254	248	248	237	220	252
≥75	1,168	1,225	1,187	1,245	1,247	1,322	1,310	1,306	1,231	1,248	1,046	946	1,201
Total	88	93	91	98	97	103	108	105	103	106	97	91	98

CHILE (2010–2020)

Age group	2010	2011	2012	2013	2014	2015	2016	2017	2018	2019	2020	Total
**Reported number of deaths due to any respiratory disease (ICD-10 codes: J00–J99)**												
18–49	395	332	295	327	320	295	330	345	354	417	333	3.743
50–64	874	766	808	848	907	937	912	953	1.037	1.252	917	10.211
65–74	1,515	1,388	1,477	1,582	1,583	1,549	1,513	1,605	1,857	2,177	1,624	17,870
≥75	7,104	6,543	7,467	7,431	7,568	7,079	7,026	7,898	8,912	9,931	7,082	84,041
Total	9,888	9,029	10,047	10,188	10,378	9,860	9,781	10,801	12,160	13,777	9,956	115,865
**Total population**												
18–49	8,251,393	8,332,294	8,407,167	8,463,306	8,516,650	8,566,336	8,624,370	8,733,996	8,899,268	9,085,578	9,264,432	95,144,790
50–64	2,588,219	2,675,332	2,762,524	2,848,603	2,935,663	3,017,783	3,090,645	3,161,019	3,234,831	3,300,485	3,359,295	32,974,399
65–74	920,338	952,502	987,847	1,024,819	1,061,928	1,104,041	1,155,332	1,211,108	1,273,583	1,335,405	1,397,997	12,424,900
≥75	678,646	701,320	724,869	749,534	775,386	802,322	830,386	859,688	891,612	924,817	960,619	8,899,199
Total	12,438,596	12,661,448	12,882,407	13,086,262	13,289,627	13,490,482	13,700,733	13,965,811	14,299,294	14,646,285	14,982,343	149,443,288
**Incidence of deaths due to any respiratory disease per 100,000 population**												
18–49	5	4	4	4	4	3	4	4	4	5	4	4
50–64	34	29	29	30	31	31	30	30	32	38	27	31
65–74	165	146	150	154	149	140	131	133	146	163	116	144
≥75	1,047	933	1,030	991	976	882	846	919	1,000	1,074	737	944
Total	79	71	78	78	78	73	71	77	85	94	66	78

MEXICO (2016–2021)

Age group	2016	2017	2018	2019	2020	2021	Total
**Reported number of deaths due to any respiratory disease (ICD-10 codes: J00–J99)**							
20–49	4,955	4,596	5,251	5,651	11,503	11,028	42,984
50–64	7,288	7,180	8,619	9,416	21,132	18,363	71,998
65–74	9,366	9,300	11,268	11,813	20,780	18,512	81,039
≥75	35,232	35,741	38,545	39,662	41,122	36,550	226,852
Total	56841	56817	63,683	66,542	94,537	84,453	422,873
**Total population**							
20–49	54,053,479	54,694,365	55,284,260	55,831,163	56,338,851	56,812,967	333,015,085
50–64	15,157,323	15,665,399	16,169,008	16,671,018	17,169,851	17,664,729	98,497,328
65–74	5,058,887	5,242,457	5,442,326	5,656,752	5,885,647	6,128,798	33,414,867
≥75	3,278,662	3,377,424	3,478,481	3,584,626	3,696,584	3,814,731	21,230,508
Total	77,548,351	78,979,645	80,374,075	81,743,559	83,090,933	84,421,225	486,157,788
**Incidence of deaths due to respiratory disease per 100,000 population**							
20–49	9	8	9	10	20	19	13
50–64	48	46	53	56	123	104	73
65–74	185	177	207	209	353	302	243
≥75	1,075	1,058	1,108	1,106	1,112	958	1,069
Total	73	72	79	81	114	100	87

MALAYSIA (2010–2022)

Age group	2010	2011	2012	2013	2014	2015	2016	2017	2018	2019	2020	2021	2022	Total
**Reported number of deaths due to any respiratory disease (ICD-10 codes: J00–J99)**														
20–49	1,552	1,423	807	1,027	1,163	1,321	1,339	1,231	1,511	1,678	1,639	3,468	1,733	19,892
50–64	2,089	2,278	1,381	1,784	2,131	2,442	2,489	2,499	3,083	3,184	2,917	5,524	3,398	35,199
65–74	2,267	2,293	1,442	1,875	2,289	2,626	2,673	2,650	3,291	3,409	2,915	4,983	3,922	36,635
≥75	2,435	3,004	1,636	2,385	2,992	3,491	3,719	3,562	4,211	4,253	3,539	5,094	5,201	45,522
Total	8,343	8,998	5,266	7,071	8,575	9,880	10,220	9,942	12,096	12,524	11,010	19,069	14,254	137,248
**Total population**														
20–49	13,243,900	13,657,800	14,043,300	14,395,400	14,711,400	14,987,000	15,218,700	15,436,700	15,637,200	15,823,700	15,998,500	16,186,500	16,356,600	195,696,700
50–64	3,260,300	3,415,600	3,565,100	3,711,000	3,855,500	3,999,000	4,139,000	4,280,800	4,421,400	4,558,200	4,690,700	4,820,100	4,947,600	53,664,300
65–74	947,300	999,500	1,056,000	1,115,700	1,180,400	1,249,900	1,323,100	1,401,400	1,482,800	1,566,400	1,652,400	1,739,500	1,827,600	17,542,000
≥75	477,800	496,300	524,900	557,100	589,700	620,700	650,400	679,500	710,200	746,100	790,300	841,700	900,700	8,585,400
Total	17,929,300	18,569,200	19,189,300	19,779,200	20,337,000	20,856,600	21,331,200	21,798,400	22,251,600	22,694,400	23,131,900	23,587,800	24,032,500	275,488,400
**Incidence of deaths due to respiratory disease per 100,000 population**														
20–49	12	10	6	7	8	9	9	8	10	11	10	21	11	10
50–64	64	67	39	48	55	61	60	58	70	70	62	115	69	66
65–74	239	229	137	168	194	210	202	189	222	218	176	286	215	209
≥75	510	605	312	428	507	562	572	524	593	570	448	605	577	530
Total	47	48	27	36	42	47	48	46	54	55	48	81	59	50

ICD-10, International Classification of Diseases 10th revision
